# From burnout to balance: the role of peer-assisted learning in college life

**DOI:** 10.1186/s12909-025-08399-7

**Published:** 2025-12-28

**Authors:** Natalia Jimenez, Isabel C. Gomez, Eduardo J. Ruiz, Ana Moreira, Legier V. Rojas

**Affiliations:** 1 Department of Interdisciplinary Sciences, University of Puerto Rico at Río Piedras, San Juan, Puerto Rico; 2 School of Medicine, Medical Sciences Campus, University of Puerto Rico (URP), San Juan, Puerto Rico; 3 School of medicine, Physiology Department, Universidad Central del Caribe, Bayamon, Puerto Rico; 4 School of Psychology, Faculdade de Ciências e Tecnologia, Universidade Europeia, Lisbon, Portugal

**Keywords:** Academic burnout, Peer-Assisted learning, Informal Peer-Assisted learning, School burnout inventory.

## Abstract

**Background:**

Academic burnout (ABO) poses a significant threat to student well-being and performance, particularly among premedical undergraduates. While informal peer-assisted learning (IPAL) may mitigate this burden, limited research has explored this relationship in nonmedical student populations. The objectives of this study are to assess the psychometric validity of the SBI-9 in the undergraduate population and to investigate whether a relationship exists between ABO and IPAL in this population.

**Methods:**

We conducted a cross-sectional survey of 245 undergraduate students at the University of Puerto Rico, Río Piedras Campus. ABO was measured using the nine-item School Burnout Inventory (SBI-9). IPAL engagement was assessed through a single-item measure. Internal consistency, item correlations, and confirmatory factor analysis (CFA) were performed to validate the SBI-9. ABO levels were analyzed by age, gender, academic year, study preference, and IPAL engagement.

**Results:**

The SBI-9 demonstrated high internal consistency (Cronbach’s α = 0.872) and a validated three-factor structure. Overall, ABO levels increased slightly across academic years, with the highest scores observed in fifth-year students. Female students reported significantly higher ABO than males, particularly in the first two years. Globally, students who never engaged in IPAL reported significantly higher ABO scores (mean = 48.41) compared to those with occasional or frequent IPAL engagement (mean = 42.48, *p* = 0.0384). A similar trend was observed in students who preferred studying alone.

**Conclusions:**

Informal peer-assisted learning may serve as a protective factor against academic burnout among undergraduate students. Gender differences and study habits further influence ABO vulnerability. Early peer-based interventions may promote academic resilience and psychological well-being in premedical populations.

## Background

Academic burnout (ABO) is a growing concern in higher education, closely linked to poor academic performance, mental health deterioration, increased dropout rates, and even suicidal ideation [1, 2]. Characterized by emotional exhaustion, depersonalization, and a diminished sense of accomplishment, ABO has been shown to negatively predict academic achievement, including performance on exams, course grades, and overall GPA [3, 4]. Addressing ABO is therefore essential to safeguarding students’ academic success and overall psychological well-being.

The nine-item School Burnout Inventory (SBI-9) is a widely used and validated instrument for assessing ABO, encompassing three core dimensions: exhaustion, cynicism, and inadequacy [5–8]. These dimensions are conceptually aligned with established models such as the Maslach Burnout Inventory [9]. They also reflect the World Health Organization’s ICD-11 classification of burnout as an occupational phenomenon resulting from chronic, unmanaged workplace stress [10]. As such, the SBI-9 provides a robust framework for assessing burnout in educational settings.

As ABO gains increasing attention in higher education research, recent studies have reported a high prevalence among university students, driven by a combination of academic and psychosocial stressors [11], with lasting repercussions for students’ educational and professional trajectories [12]. This burden is especially pronounced among students in health-related disciplines. In the United States, burnout among medical students increased by 6% between 2008 and 2014 [13], and globally, nearly half of all medical students experience ABO before entering residency training [14]. Similarly, at the Universidad Central del Caribe (UCC), local data reflect this global trend: burnout prevalence among first-year medical students rose from 10.91% in 2017 to 41.34% by 2022, with second-year students showing consistently elevated levels [7, 8, 15].

While these findings underscore the urgency of addressing burnout in medical education, emerging evidence suggests that the psychological toll of academic pressure may begin even earlier. Among premedical students, elevated levels of depressive symptoms and burnout have been linked to reduced interest in pursuing medical careers, particularly among women [16]. Furthermore, everyday stressors in this population (such as academic procrastination and test anxiety) are associated with heightened suicidal ideation, especially when combined with perfectionism and limited psychological coping resources [17].

Despite these concerning trends, research on ABO in Puerto Rico has focused almost exclusively on medical students [7, 8, 15], leaving a gap in our understanding of ABO among university students preparing for medical careers. This is particularly concerning given the established link between burnout and unprofessional behaviors in students and future healthcare providers [18]. Collectively, these findings indicate that the burden of ABO can manifest well before entering medical school, underscoring the need for early identification and targeted support within premedical populations. Therefore, there is an urgent need to examine the onset of burnout earlier in the academic timeline and identify potential protective factors that may mitigate its impact during the premedical stage.

One such protective factor is the learning environment itself. Studies have shown that a poor educational environment is strongly associated with higher levels of burnout [1, 2]. Within this context, Peer-Assisted Learning (PAL) has emerged as a promising approach to support academic success and student wellness [19–23]. Informal Peer-Assisted Learning (IPAL), in particular, refers to student-led, organically formed collaborative learning through peer interactions and study groups. While less structured than formal PAL programs, IPAL promotes knowledge exchange, social support, and the development of coping skills and self-efficacy, key contributors to academic engagement and psychological resilience. Among medical students, IPAL has been associated with reduced levels of ABO and improved academic confidence, comprehension, and problem-solving abilities [7, 8, 24].

While PAL’s benefits in medical education are documented, little is known about its impact on burnout among general undergraduate populations, particularly premedical students. Understanding how IPAL engagement may influence ABO could inform the design of low-cost, peer-driven interventions aimed at reducing academic stress and promoting student success.

This study investigates two research questions: (1) whether the SBI-9 functions as a valid and reliable instrument for assessing ABO in this population, and (2) whether a significant correlation exists between ABO and engagement in IPAL. The primary objectives are, first, to establish the validity and reliability of the SBI-9 within this specific context and, second, to address a critical gap in the literature by evaluating ABO among undergraduate students and examining its association with IPAL engagement.

We propose the following hypothesis:

- Hypothesis I: Based on the School Burnout Inventory (SBI), a three-factor model with moderate intercorrelation patterns among the components (exhaustion at school, cynicism toward the meaning of school, sense of inadequacy at school) as well as a second-order model to assess overall school burnout is expected to fit well the data.
- Hypothesis II: Informal Peer-Assisted Learning (IPAL) is strongly related to overall ABO and the three dimensions of the SBI.

By identifying modifiable factors associated with ABO, this study seeks to inform early intervention strategies that promote academic balance and psychological well-being in university students.

## Methods

### Study design

This cross-sectional survey was conducted in January 2024 at the University of Puerto Rico, Río Piedras Campus (UPR-RP). The study aimed to assess the prevalence of ABO among undergraduate students and examine its relationship with IPAL.

### Study sample

The target population included full-time undergraduate students enrolled in the Faculty of Sciences at UPR-RP. Eligibility criteria require active enrollment and provision of informed consent. Participants were recruited to fill out the anonymous questionnaire through announcements distributed by faculty members and student organizations. A total of 245 students completed the survey. The faculty of Natural Sciences at UPR-RP enrolls 1,608 students across all disciplines included in this survey. The survey was available to the entire population using the Microsoft Office Forms online platform.

This cross-sectional study included a sample of 245 participants, representing 15.2% of the total student population. This sample size provides a 95% confidence level with an estimated margin of error of ± 5.8%. Alternatively, this sample size ensures a confidence level exceeding 91% with a margin of error of ± 5%.

The number of questionnaires answered by disciplines was Biology (28), Biology (cell-Mol) (29), Interdisciplinary Studies (37), Chemistry (18), Mathematics (29), Physics (22), Computation (25), Environmental Sci (23), Nutrition & Dietetics (16), and prefer to do not mention (18).

### Survey: measurement tools

Sociodemographic data were collected, including gender, age group, and year of study. ABO was measured using the nine-item School Burnout Inventory (SBI-9) [5], which evaluates three subscales: Exhaustion (EX), Cynicism (CY), and Inadequacy (IN). The SBI-9 was selected for its strong psychometric properties and comprehensive structure, which provide detailed insights into ABO while minimizing potential confounding factors. Each item was rated on a five-point Likert scale (1 = strongly disagree to 5 = strongly agree). This scale provided a structured framework for assessing participants’ perceptions across the three dimensions of burnout while ensuring a concise yet detailed representation of their responses on the five-point scale.

Our study is also based on the SBI-9 [25], which comprises the original nine items of the SBI [5]. The Spanish-adapted version of the SBI-9 used in this study [6] followed established international guidelines for the translation and adaptation of assessment instruments [25]. While these earlier validations typically used a six-point Likert scale, we chose a scale of 5 instead of 6. The inclusion of a neutral midpoint was considered methodologically advantageous for our relatively small population, as it reduced the likelihood of biased responses from forced choices and kept the scale easy to use, supported by psychometric literature.

IPAL was assessed with one item adapted from prior studies [7, 8] “Although I study alone, I generally explain concepts to my classmates”. This approach aims to capture informal collaborative learning behaviors among undergraduate students. Responses were recorded on a three-point scale (0 = never, 3 = occasionally, 5 = frequently). For analysis, students were categorized into two groups: those who never engaged in IPAL (NE) and those who engaged occasionally or frequently (O/F). This grouping strategy was chosen to simplify analysis while capturing distinctions in students’ engagement levels.

### Statistical analysis

This cross-sectional study used Partial Least Squares Structural Equation Modeling (PLS-SEM) to test the hypothesized model. For interpretability, visualization and get the ABO mean, each Likert-type response (1–5) was linearly rescaled to a 0-100 metric using [(Likert value-1) / (5 − 1)] × 100, so that 1 = 0; 2 = 25; 3 = 50; 4 = 75, and 5 = 100, and then averaged. This practice is common in importance-performance analyses within PLS-SEM, where indicator or latent scores are reported on a 0-100 scale to aid interpretation. The rescaling is linear and does not affect standardized PLS-SEM estimates [26]. A percentage-based (neutrosophic) response format, alongside classical Likert items [27], found comparable inferential applicability (both distributions were approximately normal), with smaller means and standard deviations for the percentage scale. Parametric analyses of Likert-type data are considered robust in educational research. For the statistical descriptive and inferential test, we used multiple comparisons under one-way ANOVA (GraphPad Prism v 10.1).

After collecting the data, it was imported into SPSS Statistics 30 and Jamovi v2.3.21.0 software. The first step was to perform descriptive statistics on the sample. To determine the best-fitting model of the SBI-9, we conducted a Confirmatory Factor Analysis (CFA) comparing four competing models with varying factor configurations. CFA was performed using AMOS Graphics 30 software for Windows. The procedure followed a ‘model generation’ logic [28], considering the results obtained interactively when analyzing their fit according to the recommendations of [26]: for chi-square (χ²/df) ≤ 5; for the Tucker-Lewis Index (TLI) > 0.90; for the Goodness Fit Index (GFI) > 0.90; for the Comparative Fit Index (CFI) > 0.90; for the root mean square error of approximation (RMSEA) ≤ 0.08 [27]; for the root mean square residual (RMSR), a lower value corresponds to a better fit; and for the Parsimony GFI (PGFI) > 0.60. With the data obtained from the CFA, construct reliability was calculated for each instrument’s dimension, with a value greater than 0.70. The average extracted variance (AVE) was estimated to test convergent validity, which should be greater than 0.50 [29]. However, values above 0.40 can be accepted if the Cronbach’s alpha value of the instrument is greater than 0.70 [30]. Finally, the discriminant validity of each factor of the instruments was also tested by comparing the square root of the AVE values with the correlation values between the factors. The square root of the AVE must be greater than the correlation value between the factors.

Consistency was assessed by calculating Cronbach’s alpha coefficient, which ranges from 0 to 1, excluding negative values . A value greater than 0.70 is considered the minimum acceptable in organizational studies. The total item correlation was also calculated, which should be greater than 0.200 . Finally, a correlation matrix was constructed to determine whether the association between the variables was consistent with the three-factor structure suggested by the confirmatory factor analysis. A significant level of 0.05 was considered.

### Ethical considerations

All methods and protocols were approved by the Institutional Review Board (IRB) of UPR-RP (CIPSHI #2324-057), ensuring full compliance with the ethical standards for research.

## Results

### Part I: validation analyses

#### Study sample

In January 2024, 245 undergraduate students from the Faculty of Natural Sciences at the University of Puerto Rico, Río Piedras Campus, participated in the study. Eligibility criteria included enrolling as a full-time undergraduate student and providing informed consent.

Of the participants, 58% identified as feminine (*n* = 142), 30% as masculine (*n* = 73), and 12% (*n* = 30) selected “prefer not to answer” / other gender identities (Table 1). Regarding age distribution, 25% of the participants were between 16 and 19 years old (*n* = 61), 67% were between 20 and 23 years old (*n* = 163), and 9% were 24 years or older (*n* = 21).

**Table 1:** Demographics characteristics of the sample

		*N*	Percentage
Gender	Female	142	58%
	Masculine	73	30%
	NM/Other	30	12%
Age range	16–19	61	25%
	20–23	163	66.5%
	24>	21	8.5%
Study year	First	14	6%
	Second	52	21%
	Third	74	30%
	Fourth	66	27%
	Fifth	37	15%
	NM	2	1%

Demographic characteristics of the sample, *N* = 245, were sampled and distributed according to gender, age, and Study year

The number of students whose ABO was equal to or superior to the mean of 50 was 99, representing 40.41% of the total sample (245).

Students from all academic years were represented: 6% were first-year students (*n* = 14), 21% second year (*n* = 52), 30% third year (*n* = 74), 27% fourth year (*n* = 66), and 15% fifth year (*n* = 37). Two students (1%) did not report on their academic year.

### Reliability and validation of the SBI-9

The SBI-9’s internal structure and reliability were evaluated before hypothesis testing. The scale demonstrated high internal consistency (Cronbach’s α = 0.872), with individual item-rest correlations ranging from 0.376 (EX3) to 0.757 (IN1) (Table 2).

**Table 2:** Reliability analysis

Scale Reliability Statistics				
	Mean	SD		Cronbach’s α
Scale	2.78	0.853		0.87
Item Reliability Statistics				
	Mean	SD	Item-rest correlation	Cronbach’s α
EX1	3.09	1.27	0.647	0.855
CY1	2.73	1.07	0.677	0.853
IN1	3.03	1.20	0.757	0.845
EX2	2.88	1.16	0.516	0.866
CY2	2.57	1.13	0.677	0.853
CY3	2.68	1.24	0.593	0.860
EX3	2.96	1.22	0.376	0.879
IN2	2.72	1.37	0.685	0.851
EX4	2.36	1.22	0.582	0.861

Items Correlation Matrix and Reliability Statistics. Data derived from Jamovi v2.3.21.0. The item reliability of the sub-parameters is presented in Cronbach’s α values only

Inter-item correlations were examined to assess construct validity. The correlation matrix (Table 3) revealed statistically significant positive correlations among most items, consistent with the proposed three-factor structure of the SBI-9. The strongest correlations were observed between IN1 and CY1 (*r* = 0.635, *p* < 0.001); IN1 and IN2 (*r* = 0.63, *p* < 0.001); and CY2 and CY1 (*r* = 0.625, *p* < 0.001). In contrast, EX3 exhibited the weakest correlations, particularly with CY1 (*r* = 0.213, *p* < 0.001) and EX1 (*r* = 0.277, *p* < 0.001), consistent with its lower item-rest correlation.

**Table 3:** Correlation matrix of the SBI-9

Correlation Matrix																	
	EX1		CY1		IN1		EX2		CY2		CY3		EX3		IN2		EX4
EX1	—																
CY1	0.47	***	—														
IN1	0.62	***	0.64	***	—												
EX2	0.45	***	0.37	***	0.40	***	—										
CY2	0.41	***	0.63	***	0.60	***	0.43	***	—								
CY3	0.39	***	0.50	***	0.52	***	0.34	***	0.53	***	—						
EX3	0.28	***	0.21	***	0.33	***	0.32	***	0.28	***	0.26	***	—				
IN2	0.57	***	0.56	***	0.63	***	0.30	***	0.52	***	0.53	***	0.22	***	—		
EX4	0.45	***	0.44	***	0.46	***	0.37	***	0.43	***	0.32	***	0.33	***	0.51	***	—

Note. *** *p* < 0.001

Items Correlation Matrix. Data derived from Jamovi v2.3.21.0. Asterisks in the correlation matrix highlight statistically significant values as detailed in the table’s footnote

### Confirmatory factor analysis and model fit

The results of confirmatory factor analysis are summarized in Table 4.

**Table 4:** Adjustment indices obtained in confirmatory factor analyses

MODEL	SBI-9	CFI	GFI	TLI	SRMR	RMSEA	RMSEA 90% CI		PGFI	X^2^/df	*p*
							Lower	Upper			
M1	1 F (CIYINEX)	0.94	0.94	0.92	0.070	0.088	0.066	0.111	0.56	2.90	< 0.001
M2	2 F-a (CYIN-EX)	0.96	0.95	0.94	0.060	0.077	0.053	0.101	0.55	2.45	< 0.001
M3	2 F-b (EXIN-CY)	0.96	0.95	0.94	0.065	0.078	0.054	0.102	0.55	2.49	< 0.001
**M4**	**3 F (CY-IN-EX)**	**0.97**	**0.96**	**0.95**	**0.056**	**0.069**	**0.043**	**0.095**	**0.65**	**2.16**	**< 0.001**

Statistical values for the Confirmatory Factor Analysis (CFA) and model fit are presented as follows. Model M1 represents a single-factor (1 F) model, where all subscales (CY, EX, and IN) are combined into a single factor. Models M2, M3, and M4 represent two-factor (2 F) models with different configurations of the subscales. In M2 (2 F-a), the CY and IN subscales are grouped into one factor, while EX remains a separate factor. In M3 (2 F-b), the EX and IN subscales form one factor, and CY remains the second factor. Model M4 (3 F) is a three-factor (3 F) model in which CY, EX, and IN are represented as three distinct factors. A model in which CY, EX, and IN are factors. 1 F represents a one-factor model, 2 F represents a two-factor model, and 3 F represents a three-factor model. 2 F three different models (a, b, and c)

*χ2*   chi-square, *df* Degrees of freedom, *CFI* Comparative fit index, *GFI* Goodness of fit index, *TLI* Tucker–Lewis’s index, *RMSEA* Root means square error of approximation, *PGFI* Parsimony GFI, *p p*-value

Model M1 represents a single-factor structure, where all subscales (CY, EX, and IN) are grouped into a single factor (CYINEX). Models M2 and M3 introduced two-factor configurations (CYIN–EX and EXIN–CY, respectively). Model M4 represents the original three-factor model (CY-IN-EX), maintaining separate factors for each subscale.

Among these, Model M4, representing the original three-factor structure of exhaustion, cynicism, and inadequacy, demonstrated the best overall fit. These results confirm the three-factor structure of the SBI-9 in this undergraduate sample.

Fit indices were as follows: Comparative Fit Index (CFI) = 0.97, Goodness Fit Index (GFI) = 0.96, Tucker–Lewis Index (TLI) = 0.95, Root Mean Square Error of Approximation (RMSEA) = 0.069, 90% CI [0.043, 0.095], Standardized Root Mean Square Residual (SRMR) = 0.056, and Parsimony GFI (PGFI) = 0.65. The chi-square test (χ²/*df* = 2.16, *p* < 0.001) supported the model’s adequacy. These results confirm the factorial validity of the SBI-9 in this undergraduate sample since all adjustment indices were found to be adequate only in the three-factor model [26]. After calculating composite reliability, values of 0.77 (EX), 0.78 (CY), and 0.77 (IN) were obtained, indicating that all factors have good composite reliability. For convergent validity, the AVE values obtained are 0.46 (EX), 0.74 (CY), and 0.64 (IN), indicating the existence of convergent validity in all factors [30]. Regarding discriminant validity, all AVE squared values are higher than the correlation between the respective factors (Table 5). These results indicate the existence of discriminant validity.

**Table 5:** Correlation between factors

	EX	CY	IN
EX	0.68		
CY	0.60***	0.86	
IN	0.65***	0.74***	0.80

Note. *** *p* < 0.001

Correlation between factors and discriminatory validity for exhaustion (EX), cynicism (CY), and inadequacy (IN)

### Part II: association analyses

#### Academic burnout in college students

This analysis included 245 undergraduate students from the Faculty of Natural Sciences at the University of Puerto Rico, Río Piedras Campus. ABO levels by age group, Table 6, and gender are presented in Table 7.

**Table 6:** Academic burnout in college students’ demographics presented by age group

Descriptives				
			95% Confidence Interval	
Age	N	ABO Mean	Lower	Upper
16–19	61	40.9	34.9	47.0
20–23	163	45.8	42.6	49.1
> 24	21	44.6	37.0	52.2

Note. The CI of the mean assumes that sample means follow a t-distribution with *N* − 1 degrees of freedom

The table presents the ABO values in college students categorized by age group and gender (upper part). For each group, the sample size (N), mean ABO percentage, and 95% confidence interval (including the lower and upper bounds) are provided

**Table 7:** Academic burnout in college students’ demographics presented by gender

					95% Confidence Interval	
Age	Gender	N	ABO Mean		Lower	Upper
16–19	Female	26	54.3	***	44.1	64.4
	Male	25	27.7	***	21.2	34.1
	PNA	10	39.4		26.4	52.5
20–23	Female	108	46.8		42.6	51.1
	Male	40	44.5		38.7	50.3
	PNA	15	42.4		32.7	52.1
> 24	Female	9	44.4		30.4	58.5
	Male	7	50.0		34.7	65.3
	PNA	5	37.2		19.4	55.1

Note. The CI of the mean assumes that sample means follow a t-distribution with *N* − 1 degrees of freedom

The groups are divided by age into the following age ranges: 16–19, 20–23, and 24 and older. By gender category, the options include female, male, and ‘prefer not to answer’ (PNA). Confidence intervals assume sample means follow a t-distribution with *N* − 1 degrees of freedom. A significant statistical difference (*p* < 0.001) is observed between males and females in the 16–19 years group

When grouped by age (Table 6) students aged 16–19 exhibited mean ABO score of 40.9 (95% CI [34.9, 47.0]); those aged 20–23 showed a mean ABO score of 45.8 (95% CI [42.6, 49.1]); and students over 24 years old had a mean ABO score of 44.6 (95% CI [37.0, 52.2]). A one-way ANOVA revealed a significant difference between age groups 16–19 females and males. Across all age groups (Table 7), female students consistently exhibited higher ABO levels compared to their male peers.

#### Academic burnout by years of study

**Fig. 1:**
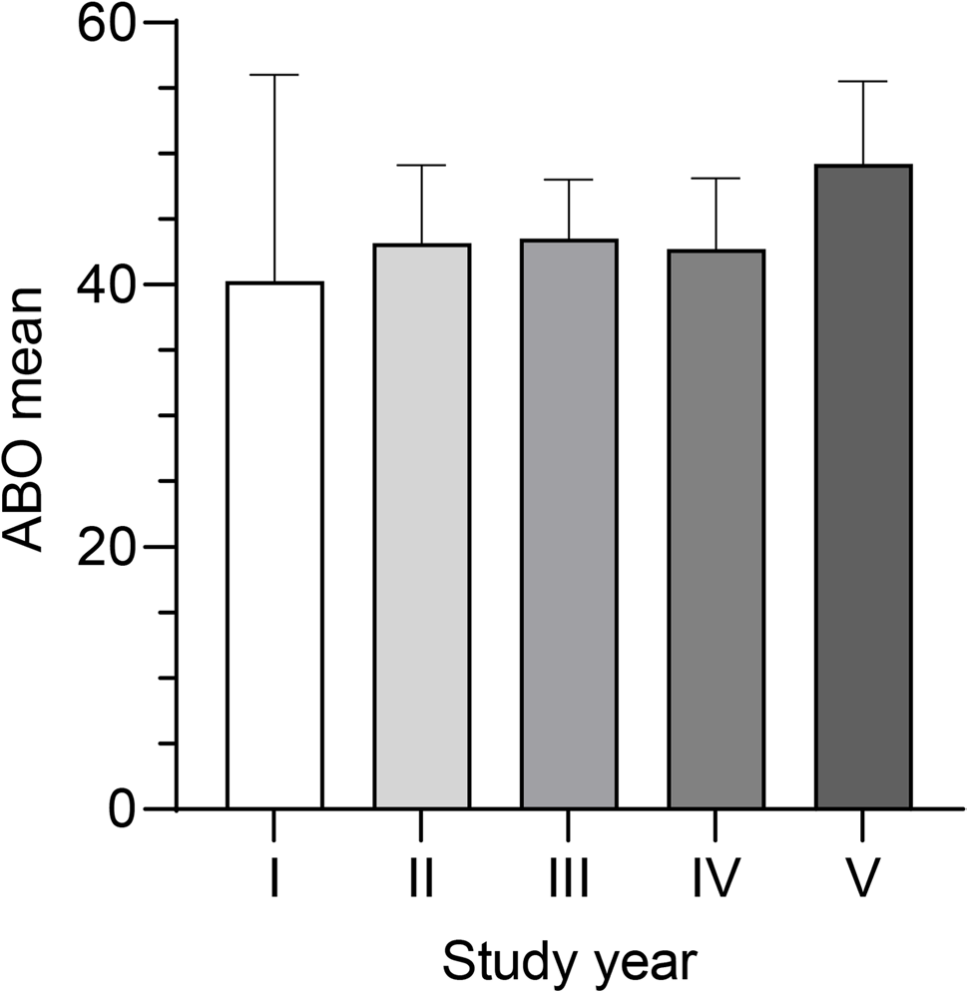
Academic Burnout Across University Years of Study. Academic burnout (ABO) levels among undergraduate university students across different years of study (*n* = 243). No statistically significant differences in ABO levels were observed across the different study years. Error bars indicate 95% confidence intervals

A one-way ANOVA was conducted to compare ABO levels across undergraduate students from the 1st to the 5th year of study (*n* = 243). The statistical analysis (Fig. 1) revealed no significant differences among groups (F(4, 238) = 0.778, *p* = 0.540, η² = 0.0129), suggesting that the year of study did not account for meaningful variation in ABO levels.

Figure 1 displays ABO levels across academic years. A trend of increasing ABO was observed from first- to fifth-year students, although differences were not statistically significant.

Figure 1 shows the mean value per study year. Mean ABO was 40.28 in 1st-year students (95% CI [24.55, 56.00], *n* = 14), 43.16 in 2nd-year (95% CI [37.21, 49.12], *n* = 52), 43.51 in 3rd-year (95% CI [38.99, 48.02], *n* = 74), 42.72 in 4th-year (95% CI [37.30, 48.14], *n* = 66), and 49.25 in 5th-year students (95% CI [42.97, 55.53], *n* = 37). Sixth-year data (*n* = 2) were excluded due to insufficient sample size. Notably, fifth-year students displayed ABO levels approaching those commonly observed among medical students, suggesting a possible cumulative effect of academic stress over time.

### Informal peer-assisted learning (IPAL) and academic burnout (ABO)

**Fig. 2:**
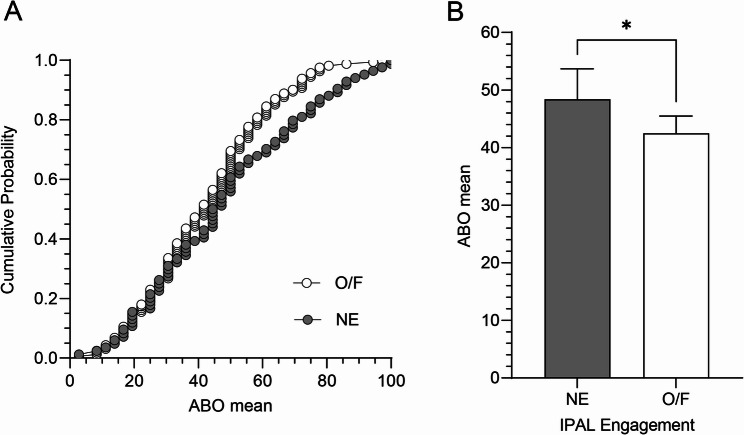
Cumulative probability distribution of academic burnout scores by informal peer-assisted learning engagement levels. Left (figure **A**), the Cumulative Probability Distribution of academic burnout (ABO) scores among medical students, grouped by their engagement in informal peer-assisted learning (IPAL). Right (figure **B**), Academic burnout (ABO) levels by engagement in informal peer-assisted learning (IPAL)

Figure 2A illustrates the cumulative probability distribution of ABO scores by IPAL engagement level. Students who never engaged in IPAL (NE, *n* = 84) reported significantly higher ABO scores (mean = 48.41, 95% CI [43.10, 53.72]) compared to those who engaged occasionally or frequently (O/F, *n* = 161; mean = 42.48, 95% CI [39.47, 45.49]). The difference (Fig. 2B) was statistically significant (*p* = 0.0384; unpaired two-tailed t-test). Error bars represent 95% confidence intervals. Total sample size: *n* = 245.

Figure 2A (left) shows that students who engaged occasionally or frequently in IPAL (O/F; white circles, *n* = 161) exhibited lower ABO scores (mean = 42.5, 95% CI [39.5, 45.5]) compared to those who never participated (NE; gray circles, *n* = 84), whose ABO mean was 48.4 (95% CI [43.1, 53.7]). In Fig. 2A, each point represents an individual student’s ABO score (*n* = 245). The distribution shows a rightward shift in burnout among students who did not engage in IPAL. Figure 2B (right) shows that Students who never participated in IPAL (NE, *n* = 84) reported significantly higher ABO scores (mean = 48.41, 95% CI [43.10, 53.72]) than those who engaged occasionally or frequently (O/F, *n* = 161; mean = 42.48, 95% CI [39.47, 45.49]; *p* = 0.0384). Error bars represent 95% confidence intervals. Total sample size: *n* = 245.

### Preferred study approach: alone or with peers

**Fig. 3:**
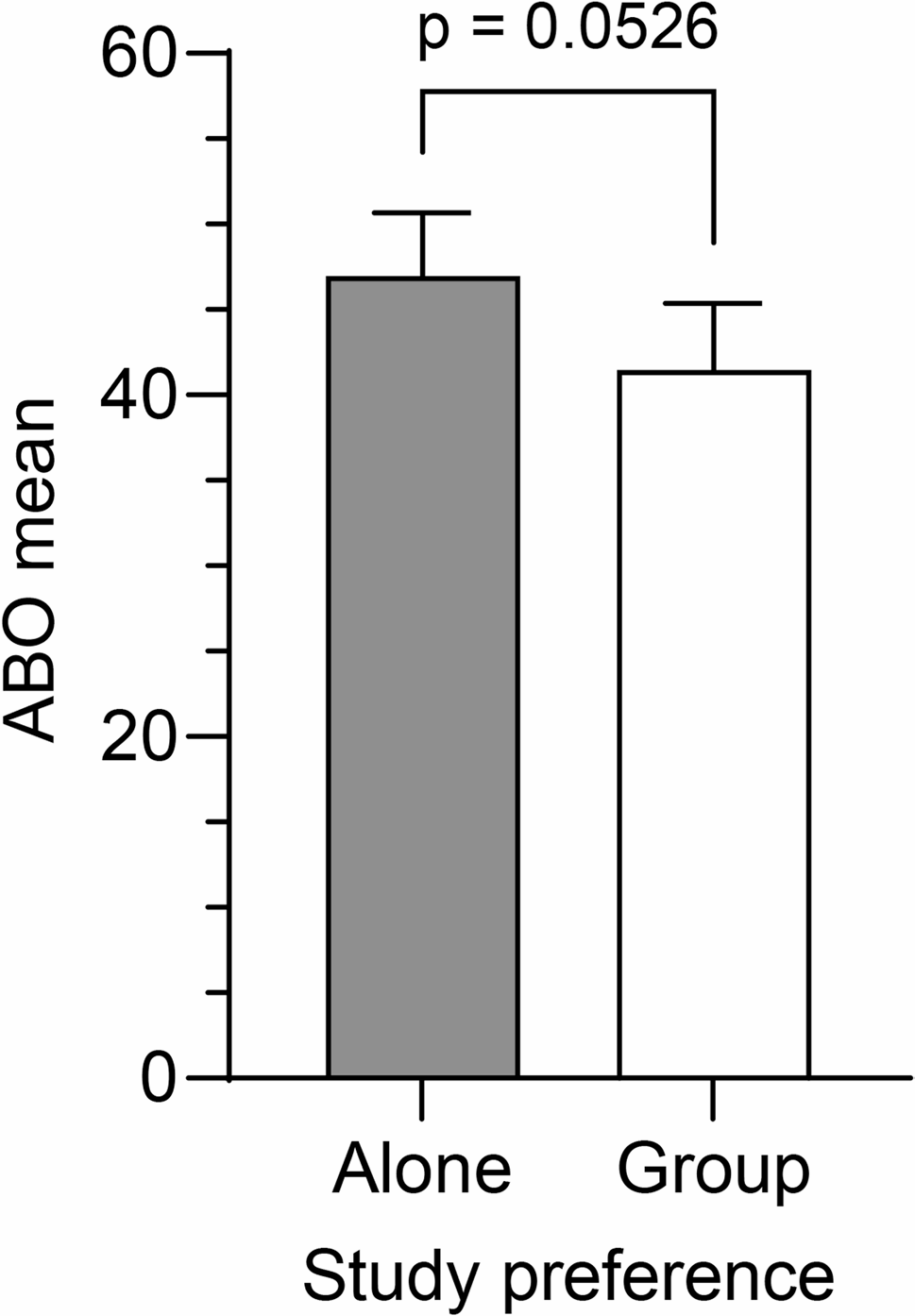
Academic burnout (ABO) levels among university students according to study preferences. Academic burnout (ABO) levels among university students according to study preferences alone and those who preferred to study with peers

In the data of Fig. 3, an unpaired *t*-test was performed, yielding *t*(235) = 1.948 with a two-tailed *p* = 0.0526. The result did not reach the conventional threshold for statistical significance (*p* < 0.05), indicating no significant difference between groups.

Figure 3 shows that students who preferred to study alone reported higher levels of academic burnout (mean = 46.97, 95% CI [43.28, 50.67], *n* = 145) compared to those who preferred studying with peers (mean = 41.49, 95% CI [37.60, 45.37], *n* = 92). While this difference approached statistical significance (*p* = 0.0526), it did not reach the conventional threshold (*p* < 0.05). Error bars indicate 95% confidence intervals. Total *n* = 237; eight students did not report a study preference.

These results suggest a potential trend toward higher burnout among students who study alone. Error bars represent 95% confidence intervals. Data from eight participants who did not indicate a study preference were excluded from this analysis (final *n* = 237).

### Gender differences in academic burnout

**Fig. 4:**
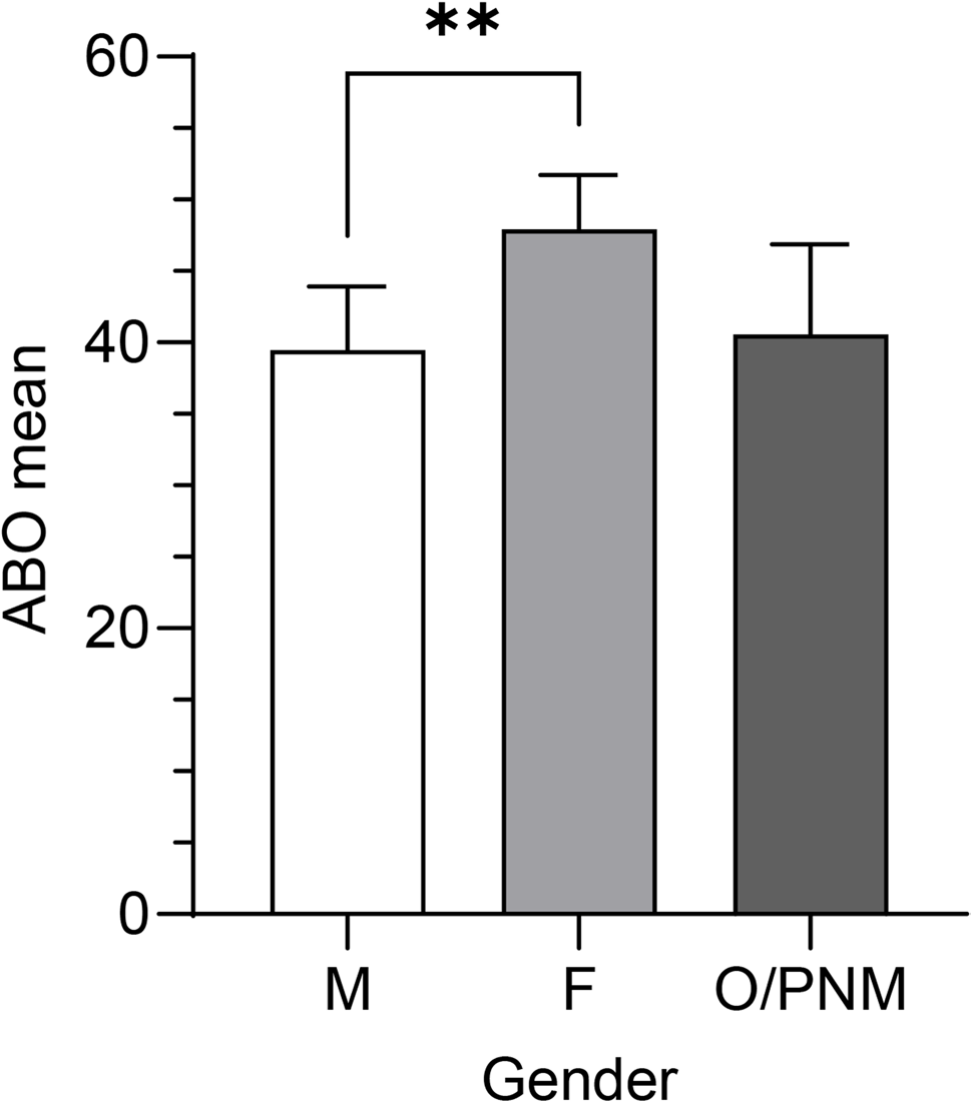
Gender Differences in Academic Burnout Among University Students. Academic burnout (ABO) levels by gender among university students. Mean ABO scores are presented for students identifying as male (M), female (F), and other/prefer not to mention (O/PNM). Error bars indicate 95% confidence intervals. Total sample size: *n* = 245

Female students reported significantly higher ABO than males (mean = 47.95, 95% CI [44.18, 51.71] *n* = 142 compared to male students having 39.46, 95% CI [35.01, 43.91]; *p* = 0.0057) (see Fig. 4). A one-way ANOVA revealed a significant difference among groups, *F*(2, 242) = 4.534, *p* = 0.0117. Students who identified as other or preferred not to specify their gender reported levels comparable to those of males, and students in the O/PNM category (mean = 40.56, 95% CI [34.25, 46.86], *n* = 30) exhibited burnout levels comparable to those of male students.

### Gender, academic burnout, and year of study

**Fig. 5:**
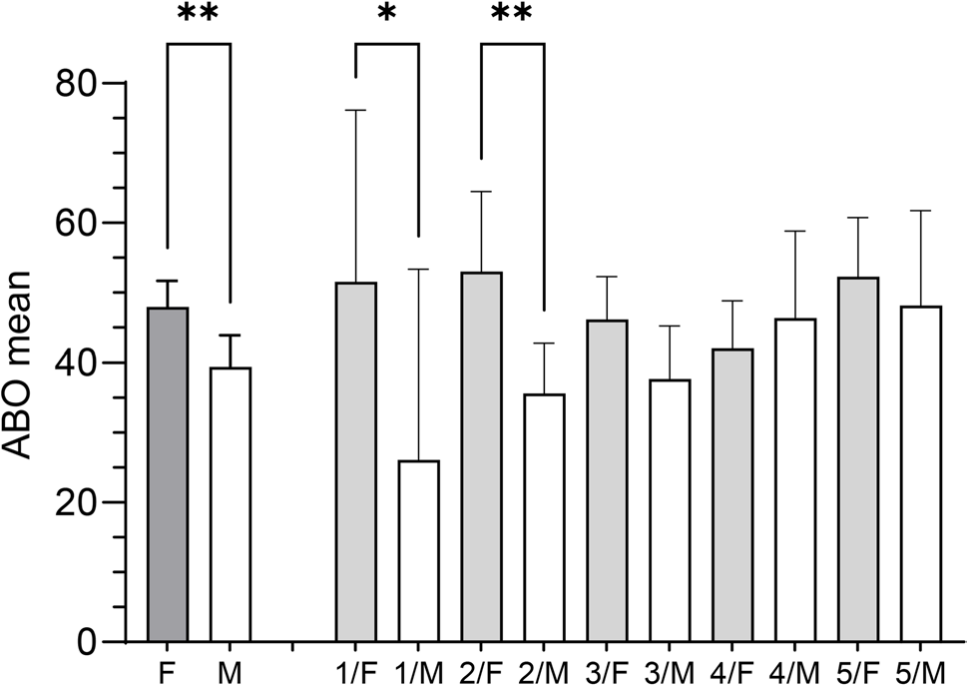
Academic Burnout by Gender and Year of Study in University Students. Academic burnout (ABO) levels among undergraduate students by gender and year of study. Error bars indicate 95% CI. Statistical significance was assessed using uncorrected Fisher’s Least Significant Difference (LSD) test

Figure 5 shows the disaggregates of ABO levels by gender and academic year. Female students (F, *n* = 142) reported significantly higher ABO levels (mean = 47.95, 95% CI [44.18, 51.71]) than male students (M, *n* = 73; mean = 39.46, 95% CI [35.01, 43.91]; *p* = 0.0057). Post hoc analyses were adjusted for multiple comparisons using the two-stage linear step-up procedure of Benjamini, Krieger, and Yekutieli (BKY) (implemented in GraphPad Software, Boston, Massachusetts, USA) to control the false discovery rate. This analysis revealed significant gender differences in both first- and second-year students. Among first-year students, females (*n* = 7) demonstrated higher ABO scores (mean = 51.59, 95% CI [27.00, 76.18]) compared with males (*n* = 5; mean = 26.11, 95% CI [− 1.11, 53.33]), reaching statistical significance (*p* = 0.0410). Similarly, among second-year students, females reported significantly higher ABO scores (*n* = 21, mean = 53.04, 95% CI [41.59, 64.49]) relative to males (*n* = 23, mean = 35.63, 95% CI [28.47, 42.79]; *p* = 0.0068). In the figure, the mean ABO scores are shown for students identifying as male (M), female (F), and other/prefer not to mention (O/PNM). Error bars indicate 95% confidence intervals. In years 3 through 5, however, ABO levels consistently trended higher among females. Notably, the highest ABO mean was observed in 2nd-year female students (2/F).

The highest average ABO values were recorded among second year (2/F) and fifth year (5/F) female students. Regression analysis of the data, plotted by year of study and gender in Fig. 5, revealed a significant positive association in males, described by the model *f(x) = 5.48x + 22.3*, with a high coefficient of determination (*r² = 0.948*) and statistical significance (*p* = 0.0051). In contrast, the model for females yielded *f(x) = − 0.995x + 51.9*, which was not statistically significant (*p* = 0.6012) and demonstrated a poor fit to the data (*r² = 0.102*).

## Discussion

This study investigated ABO among undergraduate students in Puerto Rico and examined its relationship with IPAL. The findings provide new insights into the early emergence of burnout symptoms within the premedical population, an often-overlooked group in burnout research, despite its high vulnerability to academic and psychological stress [16, 17]. The SBI-9 demonstrated strong internal consistency and factorial validity, affirming its utility as a reliable instrument in this context [5, 6]. Specifically, the CFA confirmed the original three-factor model (exhaustion, cynicism, inadequacy), indicating that the SBI-9 functions as intended in capturing the multidimensional nature of ABO in undergraduates.

Consistent with global trends, a substantial proportion of students reported moderate to high ABO levels, particularly among female students and fifth-year undergraduates [11, 14]. Although ABO levels increased slightly with the academic year, the trend did not reach statistical significance (Fig. 1). This pattern may reflect the cumulative nature of academic stress and growing uncertainty about professional futures as students approach graduation [4, 12]. Notably, the mean ABO score among fifth-year undergraduates closely mirrored those reported in Puerto Rican first-year medical students [7], suggesting that burnout may begin earlier than previously assumed and persist throughout the academic trajectory. This finding highlights the importance of monitoring student well-being throughout higher education, not just in high-stakes professional programs, such as medical studies.

A key finding was the inverse relationship between IPAL engagement and ABO. Students who occasionally or frequently participated in IPAL reported significantly lower burnout levels than those who never engaged (Fig. 2B), with an observed difference of approximately 6% points. Notably, even occasional participation appeared to be protective against burnout symptoms. This aligns with existing literature suggesting that peer collaboration enhances academic self-efficacy and buffers psychological distress [19, 21, 23]. Further supporting this trend, the cumulative distribution curve for non-IPAL students (Fig. 2A) exhibited a rightward shift, indicating consistently higher ABO scores, echoing findings from studies on medical students [8, 24]. Importantly, even after accounting for individual variability in scores, students engaged in IPAL displayed a more favorable burnout profile. These findings align with prior research emphasizing the protective role of peer interaction and academic collaboration in promoting emotional resilience. The social and cognitive scaffolding provided through IPAL may contribute to a more supportive learning environment, reducing perceived academic pressure. While causality cannot be inferred from our cross-sectional design, the consistent trend supports further exploring peer-based interventions as low-cost strategies to promote student well-being.

A non-significant yet suggestive trend further supported the benefits of collaborative learning: students who preferred to study alone had higher ABO scores than those who preferred studying with peers (Fig. 3; *p* = 0.0526). This pattern is consistent with prior findings that social academic environments can foster a sense of belonging, shared problem-solving, and emotional resilience [2, 22]. While this result should be interpreted with caution, it highlights the potential value of peer-based academic interactions in alleviating stress and promoting overall well-being.

Gender differences in burnout were also notable. Female students reported significantly higher ABO scores than males (Fig. 4). This finding supports earlier research that women often face heightened emotional demands, internalized performance expectations, and reduced access to coping resources in academic settings [1, 2]. Students identifying as non-binary or opting not to disclose their gender reported burnout levels similar to those of male students, although the small sample size limits generalizability. Nonetheless, their inclusion highlights the need for gender-inclusive mental health research. Further studies with larger, more diverse samples are necessary to explore the unique stressors and protective factors relevant to non-binary or gender-nonconforming students. Figure 5 further contextualizes gender differences by year of study. Female students consistently exhibited higher ABO scores than their male counterparts, with statistically significant differences in the first and second years. This pattern suggests that women entering university exhibit elevated burnout levels that remain relatively stable over time, with a slight decrease observed during the third and fourth years. In contrast, male students showed a more gradual and statistically significant increase across academic years, consistent with a cumulative effect of academic stress. The tendency to increase ABO in males revealed a significant positive association, described by the model *f(x) = 5.48x + 22.3*, with a high coefficient of determination (*r² = 0.948*). By later years, men’s burnout levels approached those of women. This trajectory implies that early university experiences may be particularly vulnerable periods for women due to gendered stressors, whereas for men, academic stress appears to accumulate progressively over time. Although no statistically significant gender differences were observed from the third year onward, females continued to report numerically higher ABO levels across all remaining years. These trends are consistent with existing literature linking female gender to greater emotional exhaustion, even in the absence of overt academic underperformance.

These results underscore the pressing need for early, culturally sensitive wellness strategies targeting undergraduate populations, particularly pre-medical students. Premedical students — often navigating high academic demands, perfectionism, and limited institutional support — may benefit from structured and informal wellness programs that incorporate peer-based models, such as the IPAL social intervention. These interventions could foster academic engagement and emotional well-being before burnout escalates to the more severe levels commonly observed in professional training [15–17].

Finally, the similarity in ABO levels between late-stage undergraduates and early medical students raises critical questions about the origins of burnout: Does ABO begin in medical school, or does it emerge earlier and remain unaddressed? Our findings suggest the latter, underscoring the importance of preemptive measures that extend beyond professional programs.

Academic institutions should consider promoting the social interaction offers by the informal peer-assisted learning opportunities into their educational support and wellness programs, particularly at the undergraduate and premedical levels. Fostering organic peer collaboration—such as peer study groups, tutoring circles, or mentorship networks—may help reduce the risk of burnout while strengthening academic engagement. Gender-sensitive approaches that address the unique stressors faced by female students and students from underrepresented gender identities are also warranted. By implementing early, peer-driven support systems, institutions can more effectively promote psychological resilience and academic success in future healthcare professionals.

## Limitations

Several limitations should be acknowledged. The study employed a cross-sectional design, which precludes causal interpretations. IPAL was measured through a single self-report item, which, although pragmatically informative, may not capture the full complexity of peer-learning dynamics. Additionally, the study was limited to students in the natural sciences at a single institution, which may have affected its generalizability. Finally, although gender was disaggregated beyond the binary, small sample sizes limited the robustness of statistical comparisons among non-binary or gender-diverse participants.

## Conclusions

This study contributes to the growing body of literature on academic burnout by demonstrating that burnout symptoms are already prevalent among undergraduate students pursuing premedical tracks. The validated use of the SBI-9 in this population confirms its utility as a screening tool for early detection of academic distress. Our findings suggest that IPAL may protect against burnout, offering promising, low-cost strategies to support student well-being. Furthermore, observed gender disparities and behavioral trends, such as a preference for studying alone, highlight the importance of tailoring academic wellness initiatives to student needs and lived experiences. Future research should investigate the longitudinal trajectories of burnout and assess the effectiveness of peer-based interventions across various academic disciplines and institutional contexts.

## Data Availability

The datasets used and/or analyzed during the current study are available from the corresponding author upon reasonable request.

## Abbreviations

ABO: Academic Burnout
CFA: Confirmatory Factor Analysis
CY: Cynicism
EX: Exhaustion
Fc1, Fc2 …: Factor 1, Factor 2 …
IN: Inadequacy
IPAL: Informal Peer Assisted Learning
NE: Never
O/F: Occasionally / Frequently
PCA: Principal Component Analysis
SBI-9: School Burnout Inventory -9 items
